# Inhibition of the HMGB1/RAGE axis protects against cisplatin-induced ototoxicity via suppression of inflammation and oxidative stress

**DOI:** 10.7150/ijbs.82003

**Published:** 2024-01-01

**Authors:** Xiangyun Qiao, Wen Li, Zhiwei Zheng, Chang Liu, Liping Zhao, Yingzi He, Huawei Li

**Affiliations:** 1 ENT institute and Department of Otorhinolaryngology, Eye & ENT Hospital, State Key Laboratory of Medical Neurobiology and MOE Frontiers Center for Brain Science, Fudan University, Shanghai 200031, China; NHC Key Laboratory of Hearing Medicine (Fudan University), Shanghai 200031, China.; 2 Institutes of Biomedical Sciences, Fudan University, Shanghai 200032, China.; 3 The Institutes of Brain Science and the Collaborative Innovation Center for Brain Science, Fudan University, Shanghai 200032, China.; 4 Department of Otolaryngology, Head and Neck Surgery, The Third Affiliated Hospital of Sun Yat-sen University, Guangzhou, China.

**Keywords:** Cisplatin, Hair cells, FPS-ZM1, HMGB1, RAGE, Ototoxicity

## Abstract

As an anti-tumor drug widely used in the clinic, cisplatin is limited by its ototoxic side effects associated with various factors, including inflammatory responses. Receptor for Advanced Glycation Endproducts (RAGE) recognizes damage-associated molecular patterns (DAMPs) and promotes stress and inflammation. This study intended to determine the potential behavior of the HMGB1/RAGE axis after cisplatin injury and whether it has a protective effect after inhibiting this pathway. We used FPS-ZM1, a RAGE inhibitor, to modulate the axis of HMGB1/RAGE in neonatal mouse cochlear explants and C57BL/6 mice* in vivo*. Apoptosis was identified by Annexin V-FITC/PI assay, Cleaved Caspase-3, and TUNEL staining. Reactive oxygen species (ROS) level was assessed by MitoSOX Red and CellROX Green assay. The expression of proteins associated with the HMGB1/RAGE axis and apoptosis was observed by western blotting. The expression of inflammatory cytokines was evaluated by qPCR. The protective effect of HMGB1/RAGE knockdown was also assessed on cisplatin-induced ototoxicity. These results demonstrated that cisplatin could activate the HMGB1/RAGE pathway in cochlear hair cells and release inflammatory factors. Pretreatment with FPS-ZM1 alleviated cisplatin-induced ototoxicity *in vivo* and *in vitro*. Knocking down HMGB1 and RAGE achieved specific protective effects. Altogether, inhibiting HMGB1/RAGE axis can reverse the increase of ROS accumulation, the activation of apoptosis, and the production of inflammatory reactions after cisplatin injury. FPS-ZM1 could resist the ototoxicity of cisplatin by suppressing the HMGB1/RAGE signal pathway, and it may be considered the new otoprotective potential strategy for hearing loss.

## 1. Introduction

Sensorineural hearing loss (SNHL) is the primary type of deafness, which can be caused by multiple factors such as noise over-exposure, ototoxic drugs, aging, head trauma, and genetic factors. Aminoglycoside drugs (neomycin, gentamicin, etc.) and platinum drugs (cisplatin, etc.) are the two main drugs that cause sensorineural hearing loss 1. Cisplatin, widely used in chemotherapy, can treat multiple malignancies with high side effects, namely definite ototoxicity. Ototoxicity caused by cisplatin mainly involves extensive irreversible damage to inner ear organs, including cochleotoxicity and vestibulotoxicity, thus causing bilateral progressive hearing damage 2, which causes indelible physical and mental trauma to the majority of patients relying on cisplatin therapy, especially children 3, 4.

Currently, the known targets of cisplatin ototoxicity are Corti's organs, spiral ganglia, and stria vascularis. The mechanism of refractory hearing loss caused by cisplatin may be oxidative stress, inflammation, apoptosis, and autophagy. ROS accumulation is thought to be the primary cause of cisplatin-induced hearing loss 5. However, using antioxidants as a single clinical treatment for cisplatin-induced hearing loss is not enough; it also requires strategies such as anti-inflammatory 6. Different from pathogen-induced inflammation, cisplatin-induced inflammation is a sterile inflammation resulting from acute or chronic tissue injury and does not involve the entry of pathogens 7, 8. In this circumstance, damage-associated molecular patterns (DAMPs) released by cells activated its receptors, such as pattern recognition receptors (PRRs), followed by the activation of inflammatory factors downstream 9, 10. One of the defined DAMPs is the highly conserved nuclear DNA binding protein high mobility group box 1(HMGB1), which exists in the nucleus and plays a vital role in inflammation modulation 11, 12. Receptor for Advanced Glycation Endproducts (RAGE), one of PPRs, could bind endogenous DAMPs ligands, including AGEs, HMGB1, or S100s, activate the RAGE-dependent NF-κB and mediate the inflammatory process 13, 14.

The V-domain of RAGE is the main binding area between RAGE and DAMPs 14. Several small molecule inhibitors target the RAGE-V structural domain, such as FPS-ZM1, HMGB1-derived Peptide, and Azeliragon (TTP488), which can bind to various DAMPs, including HMGB1, and have been validated in several systems 15-18. In this paper, we applied two antagonists, TFA, the peptide salt of the small peptide antagonist RAGE antagonist peptide (RAP) 19, and FPS-ZM1 17, a small molecule inhibitor. FPS-ZM1 easily crosses the blood-brain barrier (BBB). It can inhibit a series of aseptic inflammatory reactions in the brain, such as BBB damage after intracerebral hemorrhage and LPS-induced microglia inflammation 20-22. Previous studies have shown that HMGB1 accumulation in the cochlea can cause ROS accumulation and inflammation in noise-induced hearing loss 23, 24. However, it is still unclear how the HMGB1/RAGE pathway functions in cisplatin-induced ototoxicity.

Therefore, to examine whether inhibiting the HMGB1/RAGE axis significantly ameliorative effect against cisplatin-induced ototoxicity, we used FPS-ZM1 as a chemical inhibitor to experiment both in cell and animal models to provide a prospective choice for hair cell survival.

## Materials and Methods

### Cochlear explants

Cochleae from 2-day-old (P2) C57BL/6 mice were dissected and stuck to a petri dish coated with cell-Tak (BD Biosciences, USA) and then cultured in the DMEM/F12 medium, replenished with ampicillin (50 g/ml, Sangon Biotech, China; A5354-10ML) and 2% B-27 (50×, Gibco, USA; 17504-44), 1% N-2 (100×, Gibco, USA; 17502-048) overnight in an appropriate environment (33 °C, 5% CO_2_).

### HEI-OC1 cell line

HEI-OC1 cells were cultured in DMEM (Gibco BRL, USA) with high glucose and 5% FBS in an appropriate circumstance (33 °C, 5% CO_2_). The various treatments were given for subsequent experiments.

### Drug treatments *in vitro*

FPS-ZM1 (FPS, MCE, USA, HY-19370), RAGE antagonist peptide TFA (TFA, MCE, USA, HY-P2268A), D-Ribose (MCE, USA, HY-W018772) and Anisomycin (Selleck, USA, S7409) were dissolved in Dimethyl Sulfoxide (DMSO) to 10 mmol/L stock solutions. Afterward, FPS, TFA, D-Ribose and Anisomycin were diluted in the culture medium to the proprietary concentration for the experimental research and make the concentration of DMSO less than 1%. Cisplatin (Sigma‐Aldrich, USA, 479306) was stored in the 1 mM PBS reserved solution and diluted to 30 μM as the experiment concentration.

### Animal models and experimental protocols *in vivo*

According to a previous study, hearing loss associated with the combination of furosemide plus cisplatin could serve as an extremely cisplatin ototoxicity injury murine model 25. For* in vivo* experiments, the C57BL/6 cisplatin mice model was induced at P42 and P49 by intraperitoneal (i.p.) injection of cisplatin (5 mg/kg, stock in sterile saline [0.9 % NaCl]). And after 1 h injected cisplatin, furosemide (200 mg/kg) was injected intraperitoneally. FPS-ZM1 was used at a concentration of 20 mg/kg (stock in 10% DMSO+ 40% PEG300+5% Tween-80+45% sterile saline [0.9%NaCl] (The final concentration of DMSO was less than 10%.)) through i.p. 2 h before cisplatin injection. The saline injection was given subcutaneously once before and twice a day after the cisplatin treatment. After first exposure to cisplatin for 14 days, the threshold shifts of ABR (Auditory Brain Response) were measured to evaluate the degree of hearing loss. After ABR measurement, the mouse eyes were removed and bled, and the blood was allowed to clot naturally at 20°C for 20 minutes. The supernatant was extracted by centrifugation at 4°C, 2000 rpm/min for 20 minutes and the serum was collected for subsequent biochemical analysis. Mice were executed and cochleae were removed, perforated and fixed for subsequent immunohistochemistry.

### Cell viability

Cells planted in 96 well plates overnight were added to the mixture of 10 μl CCK-8 (Sigma, USA) and 90 μl media at 37 °C for 2 h. The cell viability was measured through optical density (OD) at 450 nm valued by a microplate reader (BioRad, USA).

### ROS assay

The ROS level was visualized using MitoSOX Red (Life Technologies, 1771410) and CellROX Green (Life Technologies, C10444) staining. The images were taken with confocal laser scanning (Leica Microsystems, Germany).

### Immunohistochemistry

The cochlear explants were immediately fixed overnight in 4% paraformaldehyde at 4 °C, permeabilized and blocked by 1% PBST mixed with 10% donkey serum for an hour, then incubated with primary antibodies, including anti-parvalbumin (1:500 dilution, Abcam, 32895), anti-myosin 7a (1:500; Proteus Biosciences, 25-6790), anti-cleaved caspase-3 (1:500; Cell Signaling Technology (CST), 9664s), anti-HMGB1 (1:500, ab18256), anti-RAGE (1:500, ab37647), anti-C-terminal binding protein (CtBP2, 1:1000, BD Transduction Laboratories, BD Biosciences), anti-SOX2 (1:1000, R&D Systems, Minneapolis), and anti-neurofilament (1:1000, Abcam). Cochleae were incubated for 2 h with secondary antibodies at 37 °C. And DAPI (Sigma Aldrich, D9542) labeled the nuclei. All the images were taken by confocal laser scanning (Leica Microsystems, Germany). Photoshop (Adobe Systems Inc. USA) was used to assemble, examine, and manipulate these images.

### TUNEL assay

The specimens of cochlear explants were dyed using TUNEL assay (In situ cell detection kit, Roche, 12156792910) and put in the dark for 1 h at 37 °C, and then nuclei were labeled with DAPI.

### Flow cytometry analysis for Annexin V/PI

Treated cells and their supernatants were collected, centrifuged for 5 minutes at 1000 rpm, and resuspended with binding buffer. Afterward, each group was added to 5 μl Annexin V-FITC/PI (BD Biosciences, 556547) and lightly mixed. After 20 minutes, flow cytometry was employed immediately to analyze apoptotic cells.

### Western blotting

Proteins from HEI-OC1 cells were extracted by RIPA lysis buffer containing proteinase inhibitors. The lysis lasted for 30 minutes on the ice and underwent centrifugation at 4℃, 13400 rpm for 10 minutes to collect the supernatant. 12% SDS PAGE was used to separate equal quantities of each protein sample, then diverted to PVDF membranes (Immobilon P, Switzerland) which were probed with the primary antibodies after blocking with 5% skim milk for 1 h. And secondary antibodies (1:5000 dilution; Abways, AB0101) were incubated. Protein bands were detected by Electrochemiluminescence (ECL) kit (YOBIBIO, UBI5046). All total cellular protein expression was normalized to GAPDH expression, cytoplasmic protein expression was normalized to β-actin expression, and nucleoprotein expression was normalized to Histone 3. Every experiment was repeated three times. Anti‐GAPDH-HRP (GNI, Japan, 4310-GH), anti‐β-actin-HRP (GNI, Japan, 4310-BA), anti-P38 MAPK (CST, 9212s), anti-phospho-P38 MAPK (CST, 4511s), anti-SAPK/JNK (CST, 9252s), anti-phospho-SAPK/JNK (CST, 9251s), anti-p44/42 MAPK (Erk1/2) (CST, 9107s), anti-phospho-p44/42 MAPK (Erk1/2) (CST, 4370T), anti-c-Jun (CST, 9165s), anti-phospho-c-Jun (CST, 3270s), anti-NF-κB (CST, 8242s), anti-phospho-NF-κB (CST, 3033s), anti-Bcl-2 (CST, 3498s), anti-Bax (CST, 14796s), anti-cleaved caspase-3 (CST, 9664s), and anti-caspase-3 (CST, 9662s), anti-HMGB1 (Abcam, ab18256), anti-RAGE (Abcam, ab37647), anti-IκBα (CST, 4814T), anti-phospho-IκBα (CST, 2859), anti-COX2/Cyclooxygenase 2 (Abcam, ab179800), anti-Histone 3 (CST, 4499) were used in this study. They were all diluted in the ratio of 1:500 except for GAPDH and β-actin (1:2000).

### Phalloidin staining

Cells were stained with phalloidin (1:1,000 dilution; Invitrogen-Molecular Probes, Eugene, OR, USA) for 30 minutes and then stained with DAPI for 10 minutes in the dark after being fixed and permeabilized. The F-actin and the nuclei of HC were visualized under fluorescent imaging.

### ABR test

Tests of five frequencies (4, 8, 16, 24, and 32 kHz) were given to anesthetized mice (100 mg/kg ketamine and 25 mg/kg sodium xylazine, i.p.) using TDT System III hearing aid (Tucker Davies Technologies, Gainesville, FL, USA). The hearing thresholds were recorded when the signature wave disappeared.

### Real-time PCR

Extracted RNA was transcribed into cDNA with Superscript III reverse transcriptase (Invitrogen) and amplified on the ABI 7500 real-time PCR system with TB Green (Takara). We used the β-actin gene as the endogenous control and calculated the relative expression of targeted genes with the 2-ΔΔCT formulation. The primers are demonstrated in Table 1.

### siRNA transfection* in vitro*

Designed HMGB1-specific and RAGE-specific mouse siRNAs were transfected to HEI-OC-1 cells and cochlear HCs to knock down the expression of HMGB1 and RAGE, with a non-coding siRNA as a negative control. 5 μl Lipofectamine 2000 (Invitrogen, Waltham, USA) was diluted in 200 μl Opti-MEM medium (Thermo Fisher Scientific), then added to another 200 μl Opti-MEM medium containing 100 nM siRNA. The mixed solution was placed for 20 minutes before dripping into each cell-planted plate respectively and then was replaced with DMEM containing fetal bovine serum 6 h later. 24 h cisplatin treatment was given after 24 h of transfections before cells were harvested for immunofluorescence and other assays.

### Serum oxidative stress markers assay

Superoxide Dismutase (SOD) Assay Kit (ml093072) and Malondialdehyde (MDA) Assay Kit (ml022446) were purchased from Shanghai Enzyme Link Biologicals. The collected sera were mixed with the reagents according to the manufacturer's instructions and incubated at 95°C for 1 hour to detect the SOD activity and MDA content within the sera in the different groups. The optical density (OD) was measured at 450nm (SOD) and 532nm (MDA) using a microplate reader.

### ELISA

Serum levels of cytokines (TNF-α and IL-1β) were measured by enzyme-linked immunosorbent assay kits (TNF-α (Shanghai Enzyme-linked Biotechnology, YJ002095), IL-1β (Shanghai Enzyme-linked Biotechnology, YJ301814)). The serum collected as described previously were measured at 450 nm for each well in different subgroups according to the manufacturer's instructions, and the concentrations of the corresponding cytokines (TNF-α, IL-1β) were converted from the standard curve.

### Statistical analysis

Values were examined by Graphpad Prism software. One-way ANOVA analyzed multiple-group comparisons. All data are shown as mean ± SEM, and *P* < 0.05 was considered statistically significant.

## Results

### Cochlear hair cells released HMGB1 and activated inflammatory responses via the HMGB1/RAGE axis after cisplatin injury

**Figure 1:**
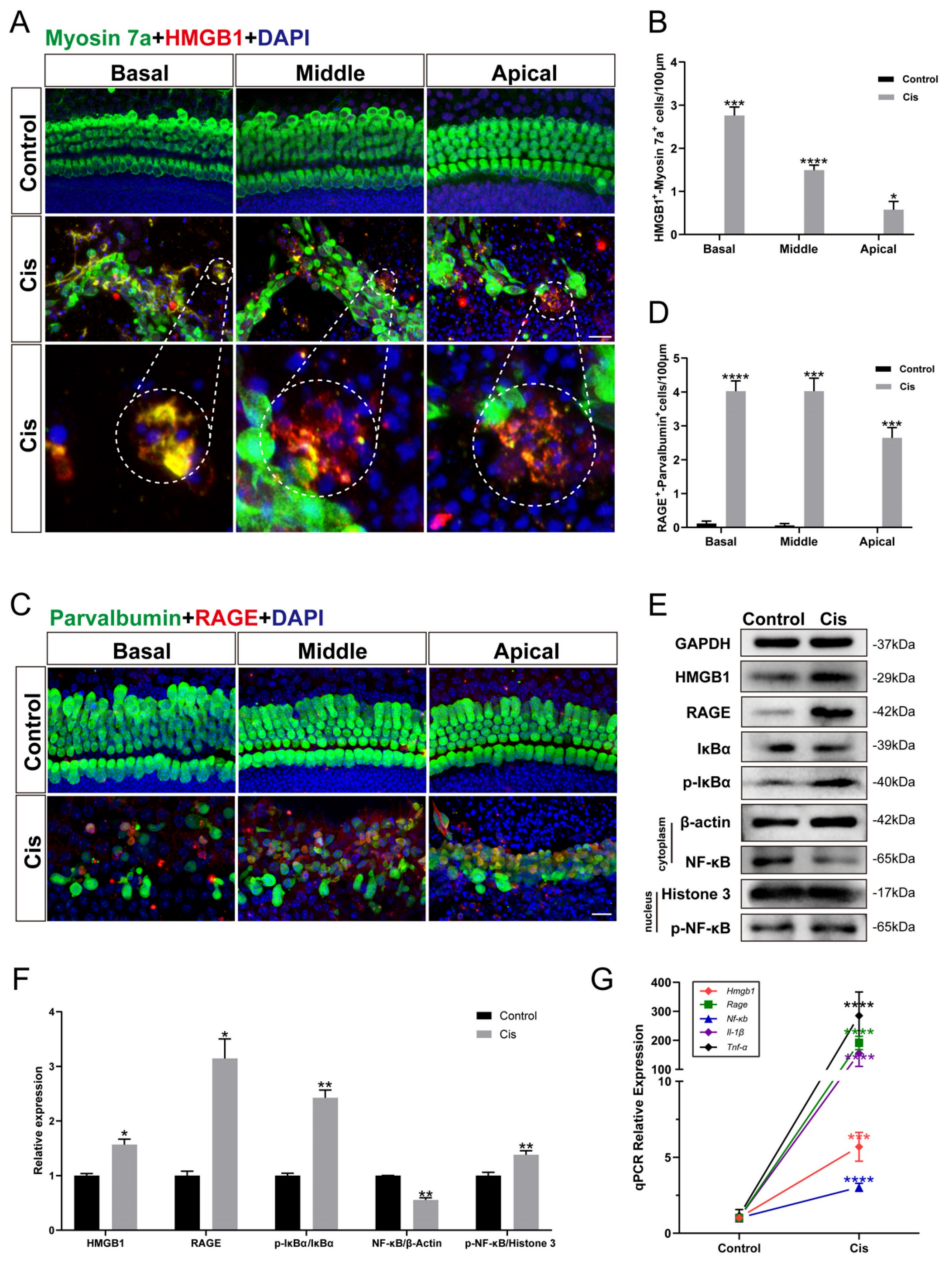
The expression changes of HMGB1/RAGE axis and activation of inflammatory factors in cochlear HCs and HEI-OC1 cells after cisplatin injury. (A) Anti-HMGB1 (red) and anti-Myosin 7a (green) co-immunostaining in the control and cisplatin groups of cochlear explants. Dashed circles were for HMGB1 release from cisplatin-induced dead HCs. Scale bar = 20 μm. (B) Statistical analysis results from HMGB1-Myosin7a double-positive cells in A (*n* = 6). (C) Anti-RAGE (red) and anti-Parvalbumin (green) co-immunostaining in the control and cisplatin groups of cochlear explants. Scale bar = 20 μm. (D) Statistical analysis results from RAGE-Parvalbumin double-positive cells in C (*n* = 6). (E) Comparison of protein expression levels in HEI-OC1 cells before and after cisplatin injury. HMGB1, RAGE, IκBα, and p- IκBα protein were normalized to GAPDH expression. NF-κB was the expression of the protein in the cytoplasm and was normalized to β-actin. P-NF-κB was the expression of the protein in the nucleus and was normalized to Histone 3. (F) Statistical analysis results of relative protein expression in E (*n* = 3). (G) The mRNA expression levels of the HMGB1/RAGE axis and inflammatory genes (*Hmgb1*, *Rage*, *Nf-κb*, *Il-1β*, *Tnf-α*) were analyzed (*n* = 3). Data are presented as the mean values ± SEM.^ *^P < 0.01, ^**^P < 0.01, ^***^P < 0.001, ^****^P < 0.0001.

To confirm whether the inflammatory response generated in cochlear HCs after cisplatin exposure is related to the activation of the HMGB1/RAGE axis, we cultured mouse cochlear explants of P2 and performed immunohistochemistry. HMGB1 and RAGE labeled themselves, and myosin 7a and parvalbumin labeled the surviving HCs. It was evident that the ruptured HCs released HMGB1 (Figure 1 A, B), and many viable cells were co-labeled with RAGE (Figure 1 C, D). Subsequently, we examined the expression of proteins in the HMGB1/RAGE axis in HEI-OC1 cells using western blotting (Figure 1 E). The obvious result was that HMGB1 and RAGE protein expressions were significantly elevated after cisplatin injury (Figure 1 F). Previous literature has suggested that RAGE-induced inflammatory response exerts its transcription factor effect mainly by phospho-IκBα. The nuclear factor NF-κB was released by its bound IκBα and phosphorylated into the nucleus 26. Our experimental results showed that IκBα significantly decreased, and p-IκBα significantly increased after cisplatin injury. Since the phosphor-NF-κB would enter the nucleus rapidly, we extracted the nuclear and cytoplasmic protein from the control and cisplatin groups separately. After cisplatin treatment, the expression of NF-κB protein in the cell plasma was decreased, while the expression of nucleus p-NF-κB protein was significantly increased (Figure 1 F). The expression of HMGB1, RAGE, NF-κB gene, and inflammation factors, such as IL-1β and TNF-α, was significantly increased in the cisplatin group versus the control group (Figure 1 G). These results demonstrated that the HMGB1/RAGE axis was significantly activated after cisplatin injury with phosphorylation of NF-κB and elevation of inflammation-related factors; thus, suppressing the pathway may be a protective method in cochlear HCs.

### The inhibitors of RAGE protected against cisplatin-induced damage *in vitro*

**Figure 2:**
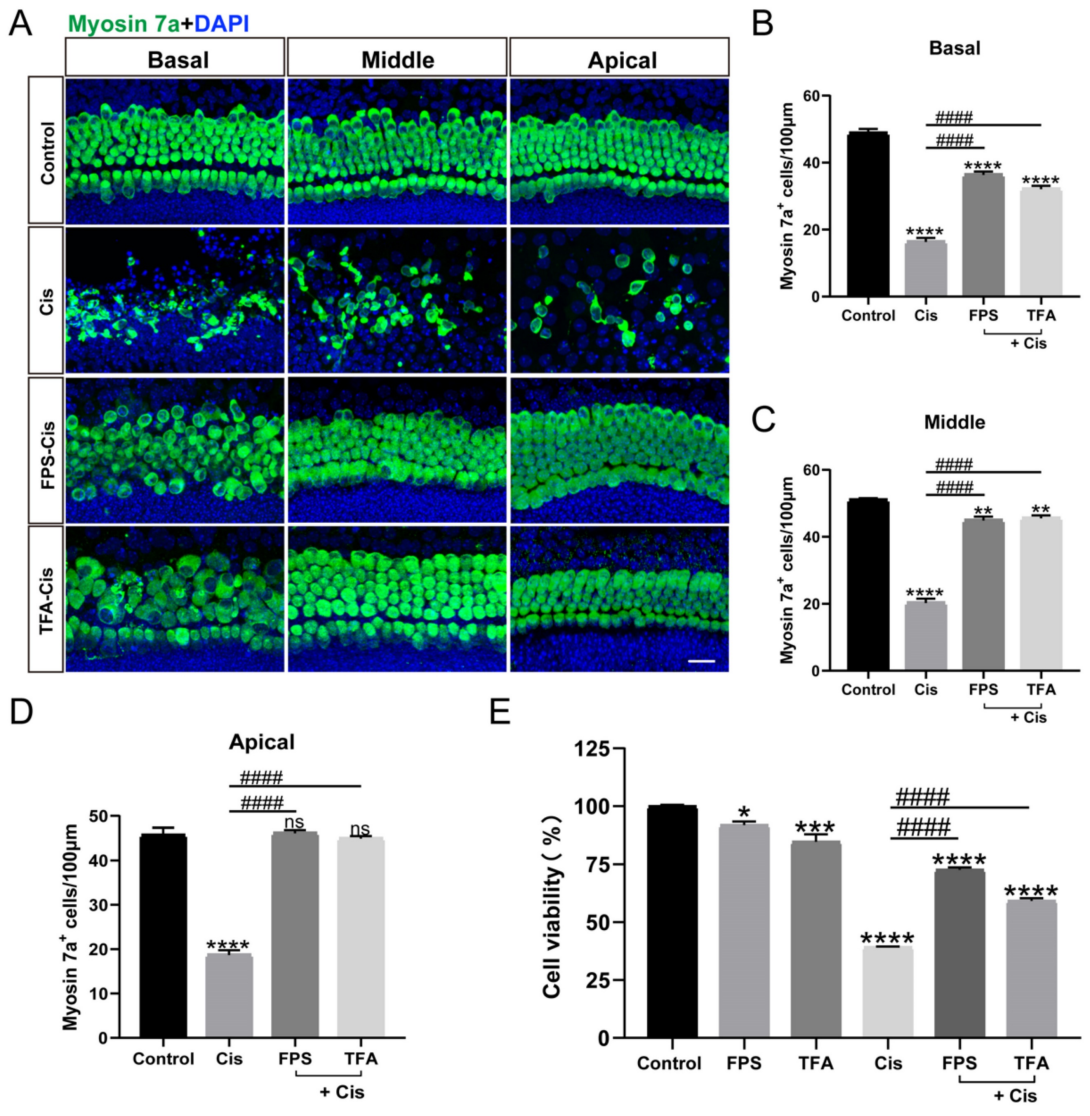
FPS-ZM1 protected HCs from cisplatin damage. (A) Representative images of cochlear HCs which were pretreated with the different inhibitors of RAGE (40 μM FPS-ZM1 (FPS) and 100 μM RAGE antagonist peptide TFA (TFA)) and co-treated with cisplatin (Cis, 30 μM). Myosin 7a (HCs, green) was used to stain HCs in apical, middle, or basal turns. Scale bar = 20 μm. (B-D) HCs were counted in each turn and statistically analyzed. Data are presented as mean ± SEM. ^**^P < 0.01, ^****^P < 0.0001 and ns. indicates no significance versus control; ^####^*P* < 0.0001 versus cisplatin-only. All the experimental groups: *n* = 6 cochlear explants. (E) The cell viability of HEI-OC1 cells was injured by 30 μM cisplatin after different RAGE inhibitor pretreatment (40 μM FPS and 100 μM TFA). Data are presented as mean ± SEM. ^*^P < 0.05, ^***^P < 0.001, ^****^P < 0.0001 versus control; ^####^*P* < 0.0001 versus cisplatin-only.

RAGE, a receptor that recognizes ligand DAMPs like HMGB1, can bind to various inhibitors such as FPS-ZM1 (FPS) and RAGE antagonist peptide TFA (TFA). To examine the effect of the inhibitors we chose, we cultured mouse cochlear explants of P2 stained with myosin 7a. From our data in Figure 2 A-D, the survival HCs pretreated with FPS and TFA increased significantly versus the cisplatin group in all turns of cochlear explants and showed significant protective effect. While in the HEI-OC1 cell line, the treatment of FPS before cisplatin showed a remarkable improvement in cell viability, and the treatment of FPS causes less cytotoxicity compared to TFA (Figure 2 E). By evaluating their protective effects in cochlear hair cells and HEI-OC1, FPS-ZM1 was subsequently used as our primary choice of RAGE inhibitor.

### FPS-ZM1 inhibited cisplatin-induced HMGB1/RAGE axis activation and inflammatory factors release

**Figure 3:**
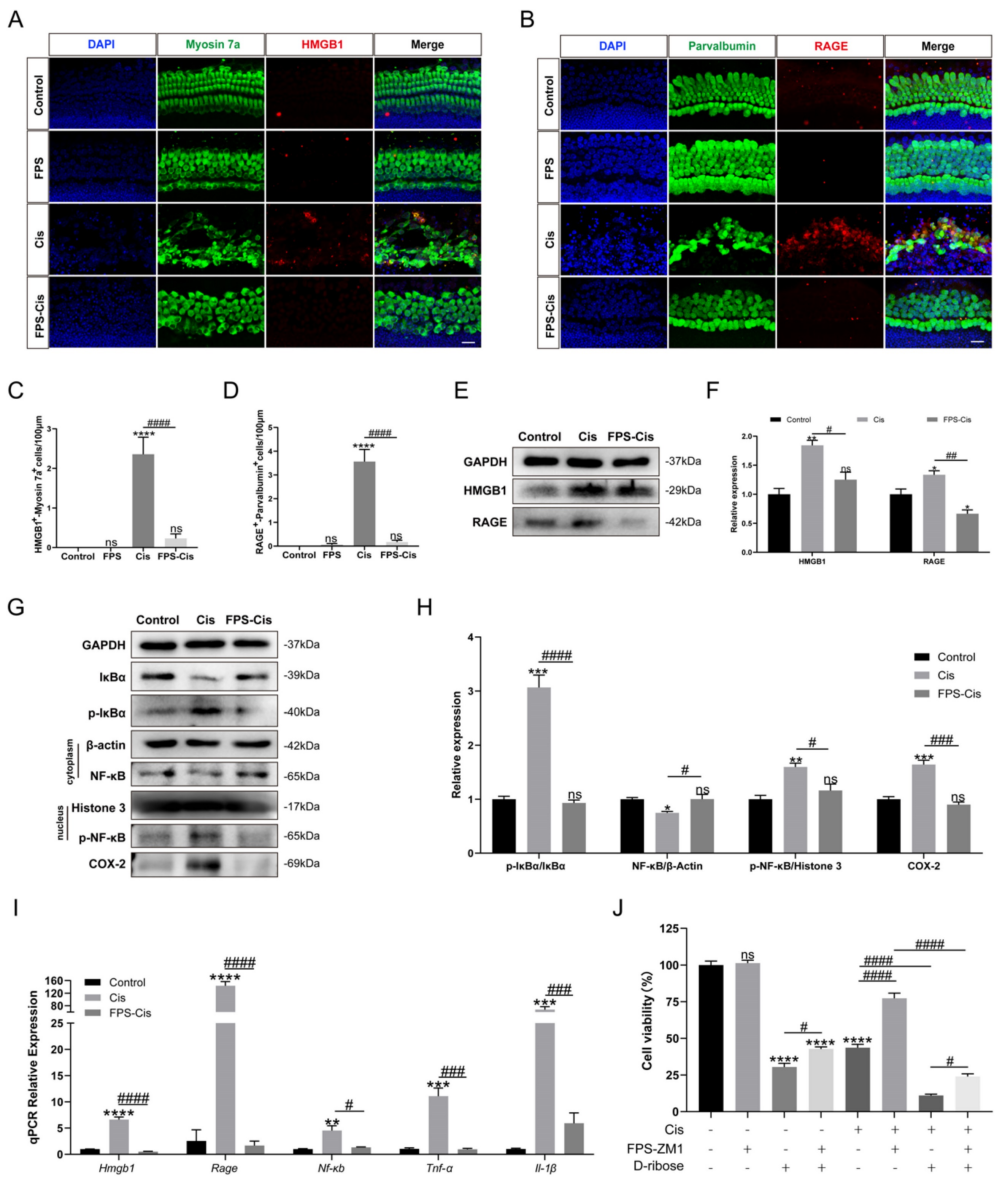
FPS-ZM1 inhibited the cisplatin-induced activation of the HMGB1/RAGE axis. (A-B) Anti-HMGB1 (red) /anti-myosin 7a (green) and anti-RAGE (red) / anti-parvalbumin (green) co-immunostaining in control, FPS-ZM1-only (FPS), cisplatin-only (Cis), FPS-ZM1 + cisplatin (FPS-Cis) groups of cochlear explants in middle turns. Scale bars = 20 µm. (C-D) Quantification of double-positive cells (HMGB1 and Myosin 7a, RAGE, and Parvalbumin). Cochlear explants of all groups: *n* = 6; (E) Comparison of protein expression levels in different groups of HEI-OC1 cells. HMGB1 and RAGE were normalized to GAPDH expression. (F) Statistical analysis results of relative protein expression in E (*n* = 3). (G) Comparison of protein expression levels in different groups of HEI-OC1 cells. IκBα, p-IκBα, and COX-2 were normalized to GAPDH expression. NF-κB was the expression of the protein in the cytoplasm and was normalized to β-actin. P-NF-κB was the expression of the protein in the nucleus and was normalized to Histone 3. (H) Results of statistical analysis of relative protein expression in G (*n* = 3). (I) The mRNA expression levels of the HMGB1/RAGE axis and inflammatory genes (*Hmgb1*, *Rage*, *Nf-κb*, *Il-1β*, *Tnf-α*) were analyzed after cisplatin exposure (*n* = 3). (J) The cell viability of HEI-OC1 cells was pretreated with different drugs (40 µM FPS-ZM1 and 20 mM D-ribose) and exposed to cisplatin. Data are presented as mean ± SEM. ^*^P < 0.05, ^**^P < 0.01, ^***^P < 0.001, ^****^P < 0.0001 and ns. indicates no significance. ^#^*P* < 0.05, ^##^*P* < 0.01, ^###^*P* < 0.001, ^####^*P* < 0.0001.

Although FPS is identified as an inhibitor of RAGE, we still need to examine the inhibitory effect of FPS in cochlear explants. Immunohistochemical staining of cochlear explants in the corresponding groups (Control/FPS/Cis/FPS-Cis) revealed a significant decrease in HMGB1-Myosin7a and RAGE-parvalbumin labeling cells when treated with FPS (Figure 3 A-D), in accordance with the expression changes of the HMGB1 and RAGE in HEI-OC1 cells (Figure 3 E-F). The trend in the downstream pathway of the HMGB1/RAGE axis also met our expectations. At protein and mRNA levels, IκBα was significantly increased, and p-IκBα was considerably decreased; NF-κB in the cell plasma remained the same, while p-NF-κB in the nucleus was remarkably reduced when treated with FPS (Figure 3G-I). Besides we also observed the inflammation-related expression in each group. COX-2, an inflammatory factor whose expression can be induced by activation of the transcription factor NF-κB, had significantly elevated protein expression after cisplatin injury, whereas COX-2 expression was significantly decreased after pretreatment with FPS-ZM1 (Figure 3 G). From the qPCR data, we concluded that FPS-ZM1 pretreatment significantly inhibited the release of IL-1β and TNF-α inflammatory factors (Figure 3 I). To confirm whether FPS-ZM1 could inhibit RAGE-induced NF-κB inflammation, we selected D-Ribose to induce NF-κB inflammation in a RAGE-dependent manner 27. We divided HEI-OC1 cells into different groups and added D-Ribose, cisplatin, or a mixture of both to cause inflammatory injury. We then found that cell viability was significantly higher in the group pretreated with FPS-ZM1, followed by the addition of D-Ribose, than in the D-Ribose alone injury group (Figure 3 J). FPS-ZM1 pretreatment also significantly antagonized inflammatory injury induced by both D-Ribose and cisplatin. These results demonstrated that FPS-ZM1 pretreated cells could prevent the activation of cisplatin-induced HMGB1/RAGE pathway, translocation of p-NF-κB into the nucleus, and generation of inflammation.

### FPS-ZM1 inhibited cisplatin-induced ROS accumulation and apoptosis

**Figure 4:**
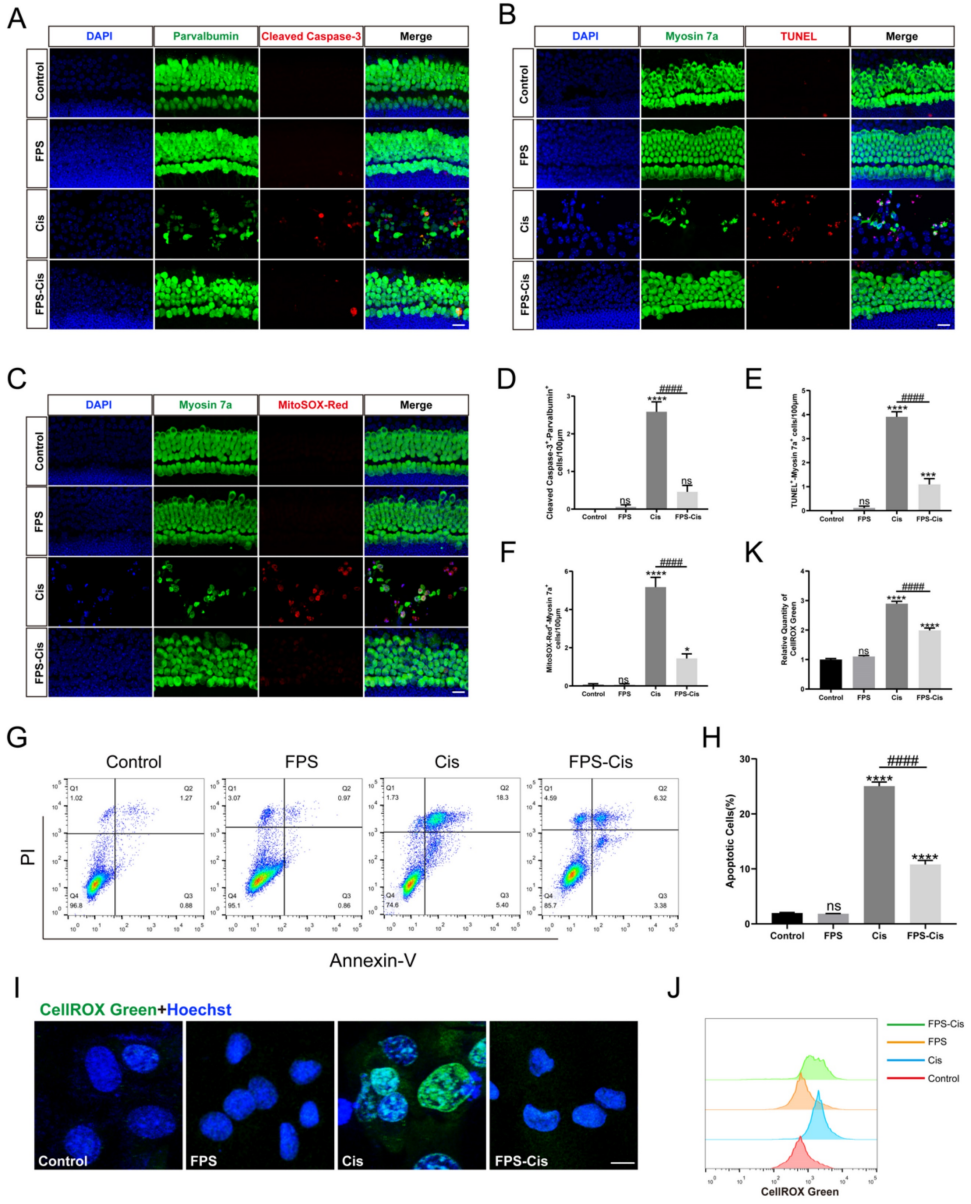
FPS-ZM1 protected against cisplatin-induced oxidative stress and apoptosis* in vitro*. (A-C) Anti-Cleaved Caspase-3 (red) /anti-Parvalbumin (green), TUNEL (red) / anti-Myosin 7a (green), and MitoSOX-Red / anti-Myosin 7a (green) co-immunostaining in control, FPS-ZM1-only (FPS), cisplatin-only (Cis), FPS-ZM1 + cisplatin (FPS-Cis) groups of cochlear explants in middle turns. Scale bars = 20 µm. (D-F) Quantification of double-positive cells (Parvalbumin and Cleaved Caspase-3, TUNEL and Myosin 7a, MitoSOX-Red and Myosin 7a). Data are presented as mean ± SEM. ^*^P < 0.05, ^***^P < 0.001, ^****^P < 0.0001 and ns. indicates no significance versus control; ^####^*P* < 0.0001 versus cisplatin-only. All the experimental groups: *n* = 6 cochlear explants. (G) HEI-OC1 cells were stained by flow cytometry using Annexin V-FITC/PI to detect apoptosis. (H) The proportion of apoptotic cells was statistically analyzed in G (*n* = 3). Data are presented as mean ± SEM. ^****^P < 0.0001 and ns. indicates no significance versus control; ^####^*P* < 0.0001 versus cisplatin-only. (I) Representative images of HEI-OC1 cells stained by CellROX Green. Scale bar = 10 µm. (J) Flow cytometry data confirmed the results in I. (K) The relative fluorescence intensity statistics of CellROX Green of J (*n* = 3). Data are presented as mean ± SEM. ^****^P < 0.0001 and ns. indicates no significance versus control; ^####^*P* < 0.0001 versus cisplatin-only.

Besides inflammation, apoptosis is the primary mode of cisplatin-induced cochlear HC death 28. To investigate whether FPS-ZM1 inhibits apoptosis apart from the inflammatory response, we used Cleaved Caspase-3 and TUNEL staining to analyze the anti-apoptotic effect of FPS-ZM1 (Figure 4 A, B). After immunofluorescence staining, we counted and analyzed double-positive cells statistically for Cleaved Caspase-3/parvalbumin and TUNEL/myosin 7a. Based on the results, we found that FPS pretreatment dramatically reduced the number of double-labeled cells after cisplatin exposure (Figure 4 D, E). We then used Annexin V-FITC/PI double staining on different conditions of HEI-OC1 cells and counted the apoptotic cells by flow cytometry in quadrants 2 and 3 (Figure 4 G, H). In our study, the percentage of apoptotic cells in the FPS pretreatment group was considerably decreased compared to the cisplatin group and showed no significant difference between the control group. These results indicated that FPS-ZM1 could dramatically reduce the increase of cisplatin-induced apoptotic cells, and it did not cause apoptosis of cochlear HCs by itself.

In the mechanism of cisplatin-induced HC injury, excessive accumulation of ROS is the key to inducing apoptosis of cochlear HCs 29. Therefore, we used MitoSOX Red and CellROX Green to assess the mitochondrial and intracellular ROS levels, respectively. In cochlear explants (Figure 4 C), MitoSOX Red/myosin 7a labeling cells were considered HCs with ROS overaccumulation, which we then counted and analyzed. We found that FPS pretreatment significantly reduced the number of labeled cells compared to cisplatin treated alone. In contrast, no significant double-positive cells were present in the FPS and control group (Figure 4 F). Meanwhile, we stained the HEI-OC1 cell line with CellROX Green and performed flow cytometry assays (Figure 4 I). Cisplatin treatment shifted the green fluorescence intensity to the right. At the same time, pretreatment of FPS turned the peak to the left side, representing a lower average fluorescence intensity (Figure 4 J-K). These above experimental results indicated that FPS could inhibit the cisplatin-induced ROS overaccumulation.

### Inhibition of cisplatin-induced inflammation and apoptosis by FPS-ZM1 was associated with inhibition of MAPK pathway activation

**Figure 5:**
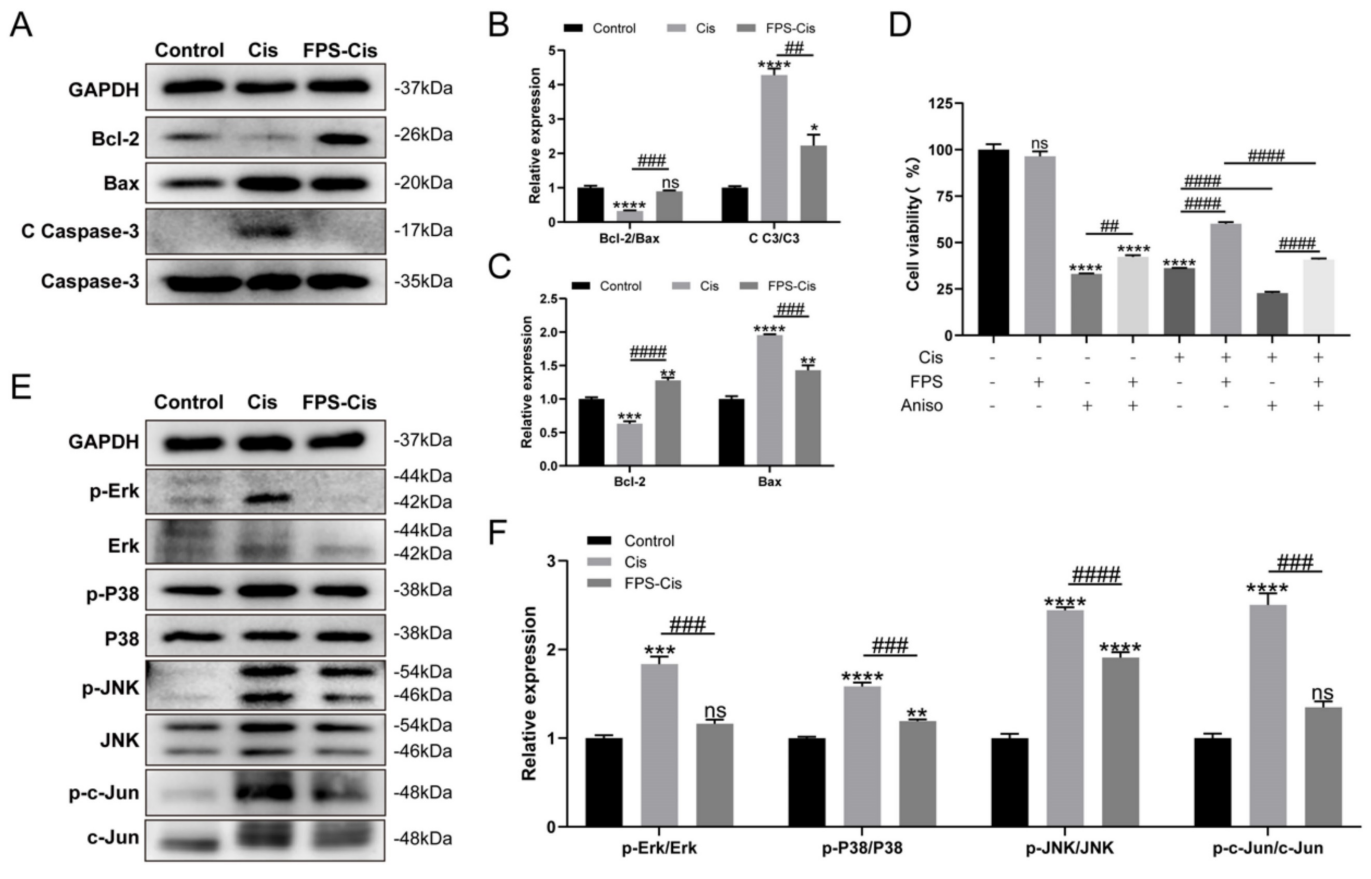
The protective effect of FPS-ZM1 was associated with the MAPK pathway. (A) Comparison of protein expression levels in different groups of HEI-OC1 cells. Bcl-2, Bax, cleaved caspase-3 (C C-3), and caspase 3 (C3) were normalized to GAPDH expression. (B-C) Results of statistical analysis of relative protein expression in A (*n* = 3). (D) The cell viability of HEI-OC1 cells was pretreated by different drugs (40 μM FPS-ZM1 and 2 μM Aniso) and exposed to cisplatin. (E) Western blotting images of the expressions of MAPKs protein: Erk, p-Erk, P38, p-P38, JNK, p-JNK, c-Jun, p-c-Jun in control, cisplatin (Cis) and FPS-ZM1 + cisplatin (FPS-Cis). (F) Protein expressions from different groups were quantified of E (*n* = 3). All protein expressions were normalized to GAPDH expression. Data are presented as mean ± SEM. ^*^P < 0.05, ^**^P < 0.01, ^***^P < 0.001,^ ****^P < 0.0001 and ns. Indicates no significance versus the control group; ^##^*P* < 0.01, ^###^*P* < 0.001, ^####^*P* < 0.0001.

From the above results, we knew that FPS could inhibit cisplatin-induced apoptosis. We examined the main ways FPS-ZM1 inhibited inflammation and apoptosis by analyzing the protein expression of HEI-OC1 cells. First, we examined several major apoptotic proteins detected by western blotting (Figure 5 A). Cleaved Caspase-3 and Bax expression was down-regulated while Bcl-2 expression was up-regulated in the pretreatment FPS group compared to cisplatin alone, suggesting that FPS could inhibit cisplatin-induced caspase-3-dependent apoptosis. We also measured the relative expression of Bcl-2/Bax between these three groups and found that pretreated with FPS inhibited the reduction of Bcl-2/Bax induced by cisplatin alone (Figure 5 B-C). These indicated that FPS could inhibit cisplatin-induced mitochondrial apoptotic pathway.

There are many ways to induce apoptosis and inflammation. It has been reported that activation of RAGE can promote Ras activation of mitogen-activated protein kinase (MAPK) signaling cascades response that inhibits IκB and thus release and activate NF-κB 30. Therefore, we examined the protein expression of MAPKs (Erk/P38/JNK) after FPS pretreatment (Figure 5 E). Figure 5 F showed that phosphor-Erk/P38/JNK protein was considerably decreased in the FPS-Cis group. To determine whether FPS-ZM1 could inhibit MAPK-induced inflammation and apoptosis, we chose anisomycin, a JNK activator, to synergistically induce apoptosis with cisplatin. We divided HEI-OC1 cells into groups by adding anisomycin, cisplatin, or a mixture of both (Figure 5 D). We found that cell viability was significantly higher in the group pretreated with FPS-ZM1, followed by adding anisomycin, than in the anisomycin-only group. Cells pretreated with FPS-ZM1 could also significantly antagonize apoptosis induced by anisomycin and cisplatin. Notably, in cisplatin injury, anisomycin blocked the cytoprotective effect of FPS, decreasing cell viability compared with the group unpretreated with anisomycin. Phosphor-JNK would phosphorylate and activate c-Jun, an activator protein-1 (AP-1) that promotes the production of inflammatory factors. Thus, we also examined the expression of phosphor-c-Jun and found that the phosphor-c-Jun was increased when treated with cisplatin and decreased when co-treated with FPS. The data above showed indicated that the MAPK pathway participated in the protective effect of FPS against cisplatin-induced hair cell injury.

### HMGB1/RAGE knockdown contributed to the survival of cochlear HCs

**Figure 6:**
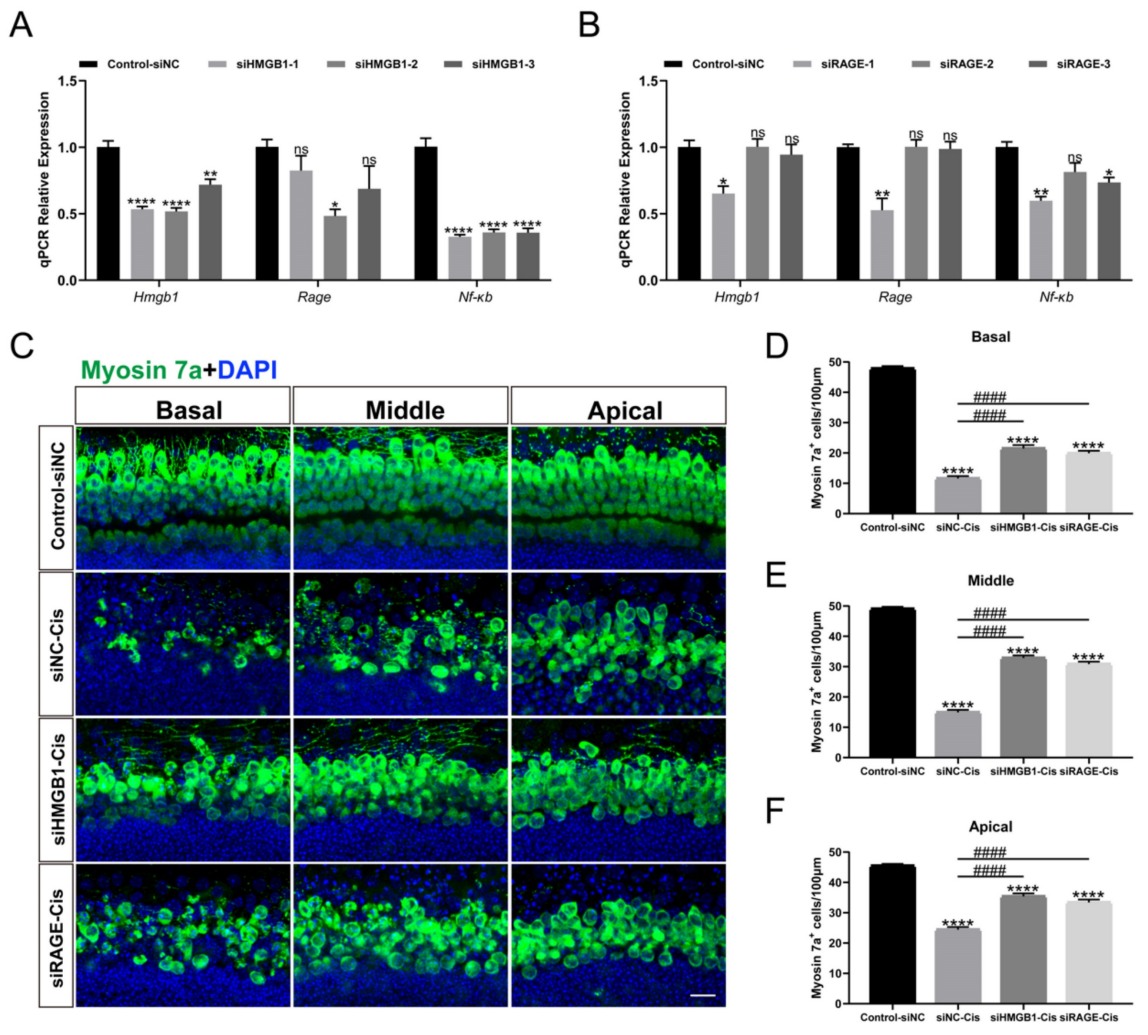
HMGB1 and RAGE knockdown inhibited cisplatin-induced HC injury. (A-B) The mRNA levels of HMGB1 (A) and RAGE (B) in siRNAs transfected HEI-OC1 cells were detected by qPCR, and values were normalized to β-actin levels (*n* = 3). Data are presented as mean ± SEM. *P < 0.05, **P < 0.01, ****P < 0.0001 and ns. indicates no significance. (C) Representative images of HMGB1 (A) and RAGE (B) in siRNAs transfected cochlear HCs. Myosin 7a (green) was used to stain surviving HCs. Scale bar = 20 μm. (D-F) Quantification of Myosin 7a-positive cells in all turns. Data are reported as mean ± SEM. ^****^P < 0.0001 versus control; ^####^*P* < 0.0001 versus cisplatin-only. All the experimental groups: *n* = 6 cochlear explants.

Transient transfection of HEI-OC1 was performed with HMGB1-specific siRNAs (siHMGB1-1, siHMGB1-2, and siHMGB1-3), RAGE-specific siRNAs (siRAGE-1, siRAGE-2, and siRAGE-3) and negative control (transfected with negative siRNA), respectively. The qPCR results showed that in siHMGB1-2 and siRAGE-1 treated cells, HMGB1 and RAGE were significantly silenced (Figure 6 A, B). Moreover, the expression of NF-κB was also considerably down-regulated after siRNA silencing, indicating that the expression of nuclear factor NF-κB was significantly inhibited after HMGB1/RAGE axis knockdown. Meanwhile, we transfected mouse cochlear explants using siHMGB1-2 and siRAGE-1 and added 30 μM cisplatin after transfection. Two days after the injury myosin 7a staining was performed, we found that in the group silenced by siHMGB1-2 and siRAGE-1, cochlear HCs had a significant protective effect in all turns compared to cisplatin-only (Figure 6 C-F). These results suggested that the downregulation of HMGB1/RAGE inhibited the cisplatin damage on hair cells.

### FPS-ZM1 inhibited cisplatin-induced hearing loss

**Figure 7:**
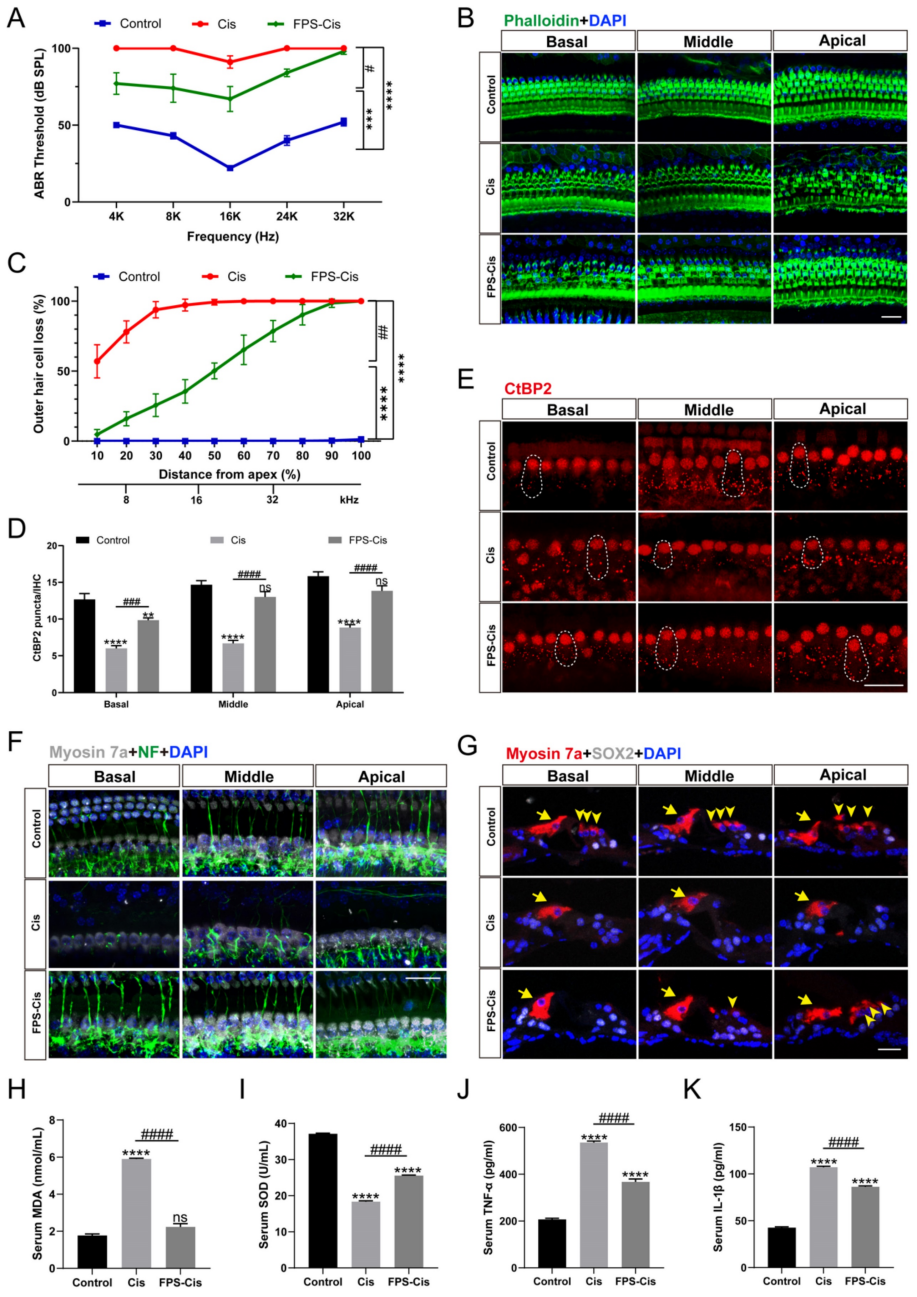
FPS-ZM1 inhibited cisplatin-induced hearing loss *in vivo*. (A) Pure-tone ABR thresholds in the control group, cisplatin-only and FPS-ZM1 + cisplatin 14 days after the cisplatin injury (*n* = 6). Data are presented as mean ± SEM. ^***^P < 0.001, ^****^P < 0.0001 versus control; and^ #^*P* < 0.05 versus cisplatin-only. (B) Representative images of immunofluorescence staining with phalloidin (green) in all turns of the cochleae from different groups. Scale bars = 20 μm. (C) Quantification of cisplatin-induced OHCs loss in different lengths from the top of the cochlea in control, cisplatin, and FPS-ZM1 + cisplatin groups (*n* = 6). Data are presented as mean ± SEM. ^****^P < 0.0001 versus control; and^ ##^*P* < 0.01 versus cisplatin-only. (D-E) Representative images of immunofluorescence staining with CtBP2 (red) in all turns of the cochleae from different groups. Scale bars = 20 μm. The statistics of CtBP2 counting in every IHC (*n* = 6). Data are presented as mean ± SEM. ^**^P < 0.01, ^****^P < 0.0001 and ns. indicates no significance versus control;^ ###^*P* < 0.001, ^####^*P* < 0.0001 versus cisplatin-only. (F) Representative images of cochlear neurofilament (NF, green), and HCs (Myosin 7a, grey) in all turns of different groups (*n* = 6). Scale bar = 20 μm. (G) Representative images of cochlear HCs (Myosin7a, red), and supporting cells (SOX2, white) in all turns of different groups (*n* = 6). The yellow arrows point to three rows of OHCs and one-row IHCs. Scale bar = 20 μm. (H-K) Serum MDA (nmol/ml), SOD (U/ml), TNF-α (pg/ml) and IL-1β (pg/ml) levels in different groups (control, cisplatin, and FPS-ZM1 + cisplatin) (*n* = 3). Data are presented as mean ± SEM. ^****^P < 0.0001 and ns. indicates no significance versus control; ^####^*P* < 0.0001 versus cisplatin-only.

C57BL/6J mice were injected with 20 mg/kg FPS-ZM1 (i.p.) 2 h before 5 mg/kg cisplatin (i.p.) to evaluate the effect of FPS-ZM1 with saline-treated mice as the control group. ABR thresholds were measured at 14 d. Figure 7 A showed that cisplatin caused significant hearing loss with a considerable increase in ABR thresholds, while pretreatment with FPS-ZM1 improved the situation (Figure 7 A), demonstrating that FPS-ZM1 protected against cisplatin-induced hearing loss. Mouse cochleae were harvested for fixed decalcification and dissected into sections from the apex to the base. Cochlear HCs were stained with phalloidin and then counted according to the length of HCs from the top to assess the loss of hair cells at different Hertz (Figure 7 B, C). FPS-ZM1 pretreatment significantly reduced the loss of HCs compared to cisplatin alone, especially in the middle and apical turns below 32 kHz. Results were the same as for staining of cochlear sections, showing the excellent protection of OHCs against cisplatin damage with FPS-ZM1 treatment (Figure 7 G). The results of this study demonstrated that FPS-ZM1 could inhibit cisplatin-induced hearing loss in mice.

Cisplatin-induced hearing loss is also associated with reduced synapses and nerve fiber damage. To check whether FPS-ZM1 can change the number of synapses, we stained the cochleae with anti-C-terminal binding protein 2 (CtBP2). The red part of each group is the CtBP2-stained presynaptic ribbons and nuclei, and the part circled by the dotted line is the synaptic dots in IHCs (Figure 7 E). Based on counting the synaptic dots inside each IHC in different groups, we obtained the following results, the mean number of anti-CtBP2 in each IHC of cochlear all turns were significantly lower after cisplatin injury versus control, and FPS-ZM1 prevented the loss of synapses caused by cisplatin (Figure 7 D). Co-immunostaining for neurofilament (NF) and Myosin 7a were carried out to find out how FPS-ZM1 functioned in the neural protection in the cochlea (Figure 7 F). Most of the axons in the control group connected IHCs to OHCs, while there was a significant depletion in the group treated with cisplatin. In the FPS-ZM1-Cis group, most nerve fibers survived and extended axons to the OHCs, showing a more significant number of NF-positive fibers. We concluded that FPS-ZM1 significantly reduced the loss of neuronal fibers and synapses induced by cisplatin.

*In vitro* experiments have shown that inflammation and severe oxidative stress are the causes of hearing loss. Therefore, we evaluated the serum of C57BL/6J mice in different subgroups. Biochemical parameters for the assessment of oxidative stress status, primarily antioxidant molecules such as SOD, and the lipid peroxidation molecule malondialdehyde (MDA). We performed orbital blood collection and collected serum from different groups of mice and measured the levels of superoxide dismutase (SOD) and malondialdehyde (MDA). The results (Figure 7 H, I) showed that serum MDA levels were significantly increased and SOD levels were significantly decreased in the cisplatin group compared with the control group. This indicated that cisplatin damage depleted serum antioxidant molecules and increased the level of serum lipid peroxidation. In the FPS-ZM1 pre-treatment group, MDA levels were significantly lower and SOD levels were significantly higher than in the cisplatin group. These results suggested that FPS-ZM1 reversed the cisplatin-induced imbalance of oxidant and antioxidant levels in serum. Similarly, we used ELISA kits to detect cytokines (TNF-α, IL-1β) levels in mouse serum, and according to the statistical results in (Figure 7 J, K), it could be seen that cytokines (TNF-α, IL-1β) levels were significantly elevated in the cisplatin group compared to the control group, and FPS-ZM1 significantly reversed the cisplatin-induced upregulation. These results demonstrated the ability of FPS-ZM1 to inhibit the increase in serum levels of pro-inflammatory cytokines induced by cisplatin.

## Discussion

The treatment of cisplatin for anti-tumor is limited by its cell resistance and severe damage to normal tissues, including ototoxicity, neurotoxicity, and nephrotoxicity 31. Several hypotheses have been proposed about the pathological mechanism of cisplatin-induced ototoxicity, and inflammation may be an essential part 32. HMGB1, as a kind of DAMPs released by damaged cells caused by aseptic trauma or pressure, can be quickly recognized and combined by PRRs, such as RAGE, and initiate the immune response to an aseptic inflammation 33. However, whether the HMGB1/RAGE axis takes part in the inflammatory response of cochlear HCs following cisplatin injury remains to be elucidated. After an in-depth study, our research verified that cisplatin-induced cell death significantly activated the HMGB1-RAGE axis. It could effectively reduce HCs damage and the production of ROS and inflammatory factors after inhibiting the HMGB1/RAGE axis. We first observed that HMGB1 was released from HCs after cisplatin injury and passed through the HMGB1-RAGE-NF-κB pathway, activating the production of downstream inflammatory factors. This study also showed that the use of inhibitor FPS-ZM1 of RAGE had a protective effect on cochlear HCs. Furthermore, FPS-ZM1 could inhibit hearing loss in adult mice, showing the reduction of ABR thresholds. Subsequently, HMGB1 and RAGE knockdown using siRNA to transfect cochlear explants also showed some protective effects *in vitro*. These results demonstrated that inhibiting the axis of the HMGB1/RAGE could be a novel cure for cisplatin-induced ototoxicity.

It has been shown in previous literature that HMGB1 major is derived from activated immune cells, necrotic cells, or injured cells 34. Although less HMGB1 is released from apoptotic cells, it can still cause significant inflammatory activation after phagocytosis by macrophages 35. Cisplatin can form DNA adducts to make HMGB1 stably bind in a double helix 36, 37. This leads to a possible anti-inflammatory capacity of non-toxic, low doses of cisplatin in the absence of apoptosis. Thus, cisplatin is protective in the mouse model of acute hepatic ischemia/reperfusion injury 38. High doses of cisplatin in the antitumor setting primarily induce apoptosis and release HMGB1 extracellularly after injury to activate a sterile inflammatory response, as has been reported in some cisplatin-induced kidney injuries 39-41. Our study used a cisplatin model that caused the severe ototoxic injury. HMGB1 release was increased after significant cochlear hair cell damage caused by cisplatin, and a clear co-labeling of damaged HCs with HMGB1 and significantly higher expression of HMGB1 proteins and mRNA could be seen in the cisplatin group. And the inhibition of HMGB1 binding to RAGE using FPS-ZM1 was able to reverse the increased HMGB1 expression and release caused by cisplatin. Subsequently, we also used siRNA to knock down HMGB1 and found that it had a protective effect on cochlear explants. Recent studies have claimed that treatment with anti-HMGB1 antibodies after noise exposure not only neutralized HMGB1 in the cochlea, reduced noise-induced hearing loss, but also reduced oxidative stress induced by noise exposure 24. These results demonstrated that reducing HMGB1 expression and inhibiting the binding of HMGB1 to RAGE could prevent cisplatin-induced ototoxic damage.

RAGE is one of the PRRs that can identify HMGB1. After verifying the expression of HMGB1 after cisplatin damage, we also examined the changes in the expression of RAGE, which significantly increased in cisplatin-induced hair cells, similarly reflected in western blotting and qPCR. Similarly, cochlear HCs appeared resistant to cisplatin damage after we knocked them down using siRAGE. According to previous literature, NF-κB is the primary signaling molecule for RAGE activation 42, 43. The NF-κB protein complex is a crucial gene transcription controller that can be isolated by κB inhibitor (IκB) in the cytoplasm and released upon IκB kinase phosphorylation 26, 44. Our results confirmed this. And we also used D-ribose, a RAGE-dependent NF-κB inflammation inducer. FPS-ZM1 was able to significantly restore the decrease in cell viability caused by RAGE-NF-κB inflammation activation. However, the pathways through which RAGE activates NF-κB are the Ras-MAPK pathway and the PI3K-PKB pathway 45. Our study focused on exploring the phosphorylation of NF-κB and the activation of the inflammatory response under the Ras-MAPK pathway. Our results confirmed that after cisplatin injury, MAPKs (JNK/p38/Erk) are phosphorylated, and all these signaling molecules may participate in the signaling mechanism of RAGE-NF-κB to induce inflammation. Notably, c-Jun, downstream of JNK, can also be phosphorylated, a member of activator protein-1 (AP-1), and can enter the nucleus to activate the transcription of pro-inflammatory genes COX-2, IL-1β, and TNF-α in concert with NF-κB, as has been already verified 46, 47. Our results also confirmed this idea.

Antioxidant drugs have been shown to provide ototoxic protection in cisplatin-treated animals 48. Sodium thiosulphate, a reducing agent, was able to reduce cisplatin-induced hearing loss after systemic administration 49. N-acetylcysteine was able to improve hearing loss in rats 50. Ebselen, a glutathione peroxidase analogue, protected against cisplatin ototoxicity and nephrotoxicity in rats when combined with allopurinol 51. The activation of inflammatory factors by RAGE may also be related to the excessive accumulation of ROS 52, 53. In the nervous system, RAGE activation facilitates mitochondrial ROS production and the secretion of inflammatory factors 54. Our results revealed that RAGE inhibitors could suppress cisplatin-induced intracellular and intra-mitochondrial ROS accumulation, balance the level of ROS and inhibit oxidative stress. Previous studies suggested that molecules with antioxidant functions (such as glutathione or heme oxygenase 1) may become critical for the RAGE activation in neurons 53, 55-57. Therefore, whether small molecule compounds with antioxidant functions have anti-RAGE effects is also worth exploring.

The damaging impact of RAGE may also be related to the apoptotic signaling exerted by RAGE. Our results validated that inhibition of RAGE inhibited caspase-3 dependent apoptosis. The expression of Bcl-2 was upregulated in the FPS-ZM1+Cisplatin group compared to the cisplatin group, which due to the fact that Bcl-2 expression is associated with protection 58. Bcl-2 is believed to act primarily in the mitochondria to prevent apoptosis 59. Previous studies have shown that Bcl-2 overexpression significantly increased the survival of SGNs deprived of nutritional support 60. Bcl-2 overexpression in SGNs promoted their recovery from ototoxic drug-induced structural damage, and concomitantly, HMGB1 was found to accumulate in the nucleus of SGNs 61. HMGB1 can positively influence SGNs' survival after ototoxic exposure through extracellular to intranuclear transfer, suggesting that the location of HMGB1 can be altered to reduce the ototoxic effects 61. It has been suggested that RAGE can mediate apoptosis by stimulating p38 MAPK and JNK 62. And from our results, we found that inhibition of RAGE activation can inhibit the JNK-induced decrease in cell viability and also have some reversal of the dual damage effect of JNK activation and cisplatin. Therefore, activation of the HMGB1/RAGE axis leading to oxidative stress and apoptosis may also be an essential pathway for cisplatin damage.

With the above experimental results, we identified and validated a new form of cisplatin damage targeting cochlear HCs: the inflammatory damage effect of activating the HMGB1/RAGE axis. And we also blocked this pathway by small molecule inhibitor FPS-ZM1 and siRNA and observed an apparent protective effect. Suppressing the HMGB1/RAGE axis could be essential for HCs survival.

## Conclusions

This study showed that suppression of the HMGB1-RAGE axis prevented the production of pro-inflammatory factors, inhibited apoptosis in cochlear hair cells, and provided a functional hearing-protective effect *in vivo*. Therefore, the modulation of the HMGB1-RAGE pathway may be a novel cure for HC survival.

## Figures and Tables

**Figure 8:**
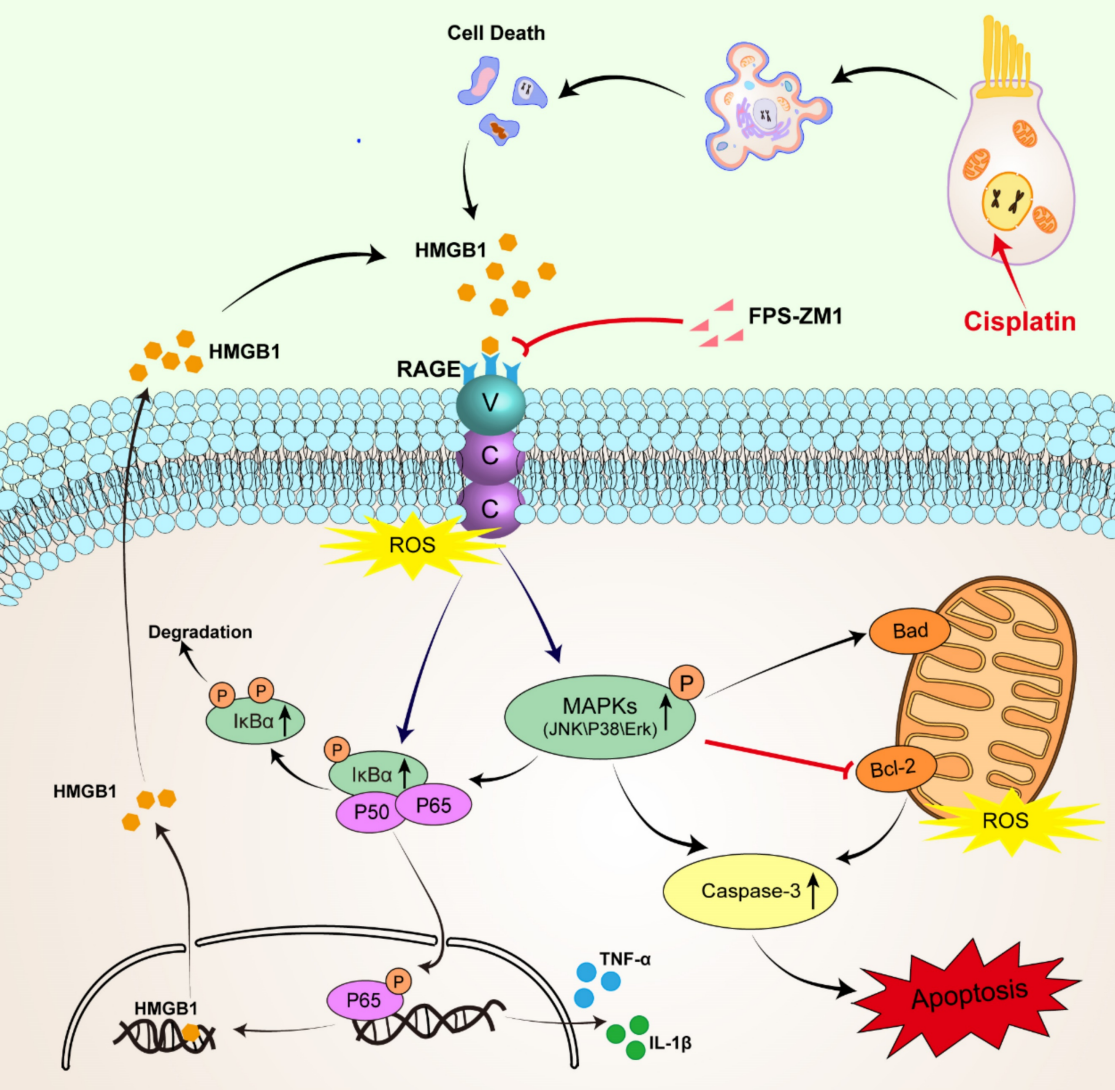
Schematic diagram of the possible beneficial mechanism of FPS-ZM1 in cochlear hair cells.

**Table 1:** Primers in qPCR

Gene	Forward Primers (5'-3')	Reverse Primers (5'-3')
*Hmgb1*	CCTAAGAAGCCGAGAGGCAA	AAGTTGACAGAAGCATCCGGG
*Rage*	CGGGACTCTTTACACTGCG	CCTTCAGGCTCAACCAACA
*Nf-κb*	GGACCTATGAGACCTTCAAGAG	ACAGAAGTTGAGTTTCGGGTAG
*Il-1β*	TCTAGGCTTTCAATGAGTGTGCC	ATCTTTTGGGGTCCGTCAACT
*Tnf-α*	GACGTGGAACTGGCAGAAGAG	TTGGTGGTTTGTGAGTGTGAG

**Table 2:** siHMGB1 and siRAGE sequence information

siRNA	Sense (5' to 3')	Anti-sense (5' to 3')
siRNA-HMGB1-1	UGACAAGGCUCGUUAUGAAAGTT	CUUUCAUAACGAGCCUUGUCATT
siRNA-HMGB1-2	GAAGAUGAUGAUGAUGAAUAATT	UUAUUCAUCAUCAUCAUCUUCTT
siRNA-HMGB1-3	GGGAGGAGCACAAGAAGAATT	UUCUUCUUGUGCUCCUCCCTT
siRNA-RAGE-1	UGGCAAAGAAACACUCGUGAA tt	UUCACGAGUGUUUCUUUGCCA tt
siRNA-RAGE-2	GAGCUGAAUCAGUCAGAGGAA tt	UUCCUCUGACUGAUUCAGCUC tt
siRNA-RAGE-3	GCAGCUAGAAUGGAAACUGAA tt	UUCAGUUUCCAUUCUAGCUGC tt

## Abbreviations

ABR: Auditory Brain Response
BBB: blood-brain barrier
CCK-8: Cell Counting Kit-8
DAMPs: Damage-associated molecular patterns
DAPI: 4',6-diamidino-2-phenylindole
DMEM: Dulbecco's modified eagle medium
DMSO: Dimethyl sulfoxide
FBS: fetal bovine serum
HCs: hair cells
HMGB-1: high mobility group box 1
IHCs: inner hair cells
IL-1β: interleukin-1β
IκBα: kappa B inhibitor
MDA: malondialdehyde
NF-κB: nuclear factor-kappa B
OD: Optical density
OHCs: outer hair cells
P: Postnatal day
PAMPs: pathogen-associated molecular patterns
PBS: phosphate-buffered saline
ROS: reactive oxygen species
RAGE: receptors of advanced glycation endproducts
SGNs: spiral ganglion neurons
SOD: superoxide dismutase
TNF-α: tumor necrosis factor-alpha
